# Prevalence and detection of *Tropheryma whipplei* in the stools of Korean patients with diarrhea using real-time PCRs

**DOI:** 10.1186/s12941-022-00543-1

**Published:** 2022-12-06

**Authors:** Sumi Yoon, Yoo Jeong Choi, Yong Kwan Lim, Oh Joo Kweon, Hye Ryoun Kim, Tae-Hyoung Kim, Mi-Kyung Lee

**Affiliations:** 1 Department of Laboratory Medicine, Chung-Ang University College of Medicine, Seoul, Korea; 2 Department of Urology, Chung-Ang University College of Medicine, Seoul, Korea

**Keywords:** *Tropheryma whipplei*, Prevalence, Stool, Korean, Diarrhea, Real-time PCR, *rpoB*, *hsp65*, *Dig15*

## Abstract

**Background:**

The prevalence of *Tropheryma whipplei* varies depending on age, region, and underlying disease. We estimated the prevalence of *T. whipplei* in the stools of Korean patients with diarrhea using real-time PCR (RT-PCR) and compared three RT-PCR targets, *rpoB*, *hsp65*, and *Dig15*.

**Methods:**

A total of 1404 nucleic acid samples extracted from the stools of Korean patients with diarrhea were tested using an initial RT-PCR targeting *T. whipplei*-specific regions of 16S–23S rRNA intergenic spacer. Subsequently, the samples positive for the initial RT-PCR were tested using the follow-up RT-PCRs targeting *rpoB*, *hsp65*, and *Dig15* and analyzed by sequencing to confirm the presence of *T. whipplei*. We estimated the prevalence of *T. whipplei* and compared them according to gender and age. We also compared the performance of three targets in the follow-up RT-PCRs.

**Results:**

*T. whipplei* was detected in 1.4% of all samples (20 of 1404), and there were no differences according to gender and age. In pediatric samples (≤ 19 years), *T. whipplei* was detected higher in children aged 6–19 than in those aged 1–5 (2.7% vs. 0.7%, *P* = 0.01). Sensitivities of the *rpoB*, *hsp65*, and *Dig15* RT-PCR were 50.0%, 85.0%, and 95.0%, respectively; specificities were 100.0%, 100.0%, and 84.6%, respectively.

**Conclusions:**

This is the first study that estimated the prevalence of *T. whipplei* in the stools of Korean patients with diarrhea. This study demonstrated the presence of *T. whipplei* in stools of Koreans, even though the bacterium was detected low. The RT-PCRs targeting *hsp65* and *Dig15* showed reliable performance, and a multiplex PCR including these targets is expected to be useful for *T. whipplei* detection.

## Background

Whipple’s disease is a rare infectious disease caused by *Tropheryma whipplei*, which has acute and chronic forms and can be fatal if not treated [1]. *T. whipplei* infections affect various organs, including the gastrointestinal tract, bones/joints, the central nervous system, and the cardiovascular system and exhibit highly polymorphic manifestations [1–3]. *T. whipplei* is a Gram-positive, rod-shaped bacterium [1, 4]. It is difficult and complicated to cultivate *T. whipplei* because it requires some supplements and there are a large number of bacteria that coexist in samples [1, 5]. For this reason, it was difficult to identify the characteristics of *T. whipplei* until it was first cultured in 1997 [5]. Whipple’s disease is commonly diagnosed by histological and molecular detection of the bacterium [1, 3]. The histological method is performed by periodic acid-Schiff (PAS) staining and immunohistochemistry on intestinal biopsy specimens [3]. It is also histologically diagnosed by confirming the presence of PAS-positive macrophages in infected lesions [1, 6]. However, the disadvantage of histological methods is that several biopsy specimens are needed because the bacterium is not evenly distributed in the intestine [1]. The PAS staining can give negative results for chronic localized infections or false-positive results for other bacterial infections [1, 7]. The molecular method is based on the polymerase chain reaction (PCR), which is more sensitive and specific than other methods [1]. A PCR targeting *T. whipplei*-specific regions of the 16S–23S rRNA intergenic spacer (ITS) and a real-time PCR (RT-PCR) targeting the repeated bacterial sequences have been developed [1, 3, 6, 8–10]. Many previous studies have used these PCR targets to detect *T. whipplei* and have demonstrated their performance [8–18]. A few commercial kits for detection of *T. whipplei* have also been developed for research purposes but not for clinical use [10].

Although the prevalence of Whipple’s disease is low, *T. whipplei* is a common intestinal bacterium because its DNA has been detected in the saliva and stools of asymptomatic carriers [14, 15]. The prevalence of *T. whipplei* varies depending on age, region, and underlying disease [14, 15, 17–21]. In Europe, its prevalence varies between 1.5% and 4% in stools of asymptomatic carriers but is higher in stools of sewer workers and patients with human immunodeficiency virus infections and liver cirrhosis [14, 19–21]. High prevalence in children has been reported at 27.5% in Ghana and 48% in Laos [18, 21]. In Korea, only three studies have reported the prevalence of *T. whipplei* [12, 22, 23]. The presence of *T. whipplei* DNA was investigated in 56 joint fluid samples of arthritic patients and in eight saliva and 22 gastric juice samples of healthy individuals; all results were negative [12]. The prevalence of *T. whipplei* was 1.9% (2 of 108) and 1.5% (2 of 132) in saliva of spondyloarthropathy patients and healthy individuals, respectively [22]; it was 1.1% (1 of 89) in saliva of 53 reflux esophagitis and 36 irritable bowel syndrome patients [23]. To the best of our knowledge, there has been no study in Korea to estimate the prevalence of *T. whipplei* in stools. In this study, we aimed to estimate the prevalence of *T. whipplei* in the stools of Korean patients with diarrhea using RT-PCRs targeting the *T. whipplei*-specific sequences used to detect the bacterium in previous studies. We also compared three RT-PCR targets, the β-subunit of RNA polymerase (*rpoB*), heat shock protein 65 (*hsp65*), and *Dig15* gene segments from Wnt1-inducible signaling pathway (WiSP) family protein (*Dig15*).

## Methods

### Study population

This study was conducted in January, 2022 in Chung-Ang University Medical Center (CAUMC), Seoul, Korea. This study protocol was approved by the Institutional Review Board (IRB) of CAUMC (2111-054-485). Informed consent from subjects was waived according to the IRB policy because the residual nucleic acid samples were used after the requested test was performed. A total of 1404 nucleic acid samples were collected, which were extracted from the stools of Korean patients (654 females and 750 males; median age (range), 7 (1–100) years) with diarrhea who visited CAUMC between January 2017 and December 2019. None of the patients were diagnosed with Whipple’s disease at the time of hospital visit or hospitalization, and thereafter. The data were analyzed anonymously.

### Molecular assay

Nucleic acids were extracted from the stools within 24 h of collection and storage at 4 °C using the NucliSENS easyMAG system (bioMérieux SA, Marcy l’Etoile, France) according to the manufacturer’s instructions. The nucleic acid samples were stored at −70 °C until used.

**Fig. 1:**
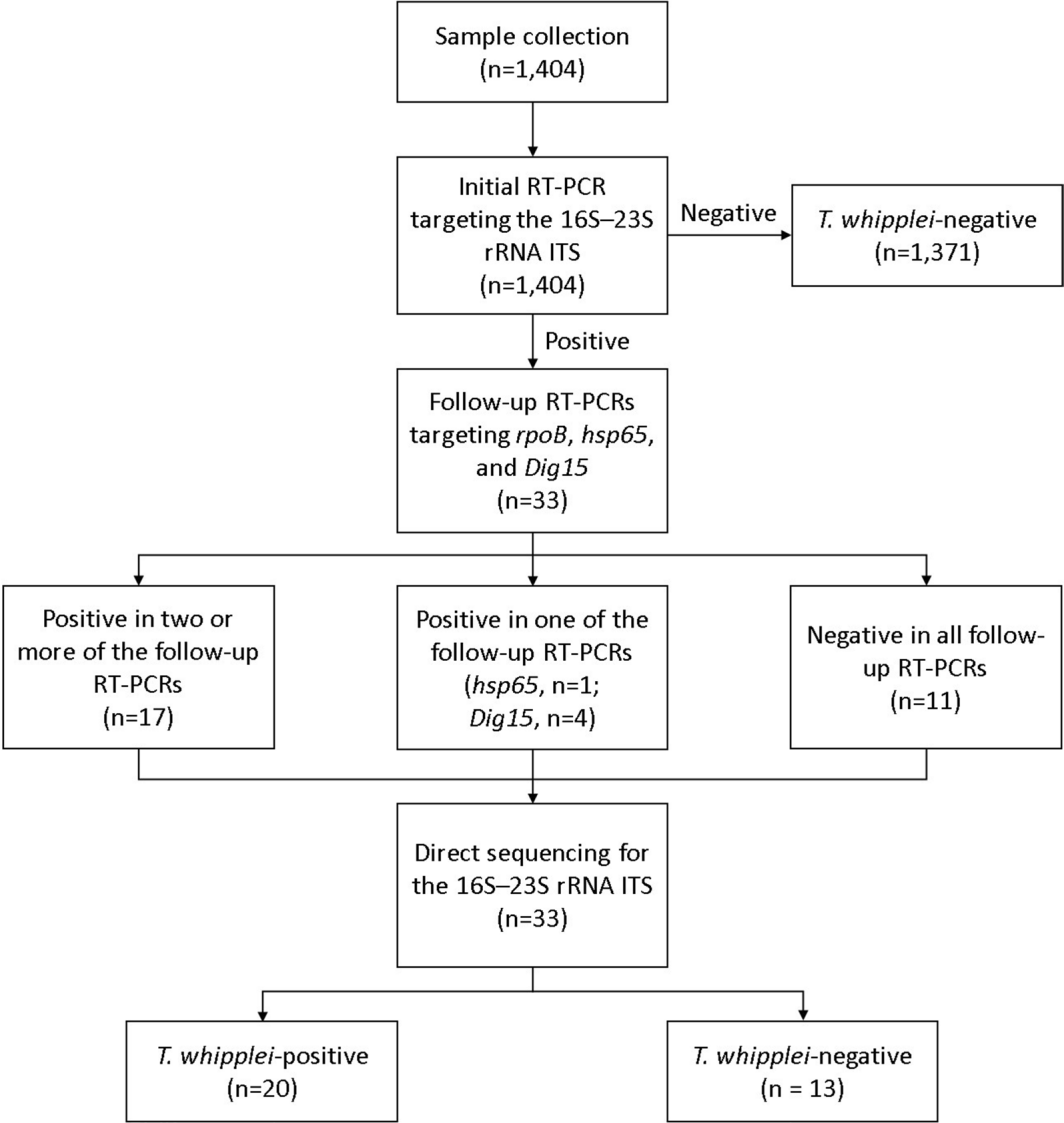
Study flow chart. *ITS* intergenic spacer, *RT-PCR* real-time PCR, *T. whipplei*, *Tropheryma whipplei*

All RT-PCRs were performed on an ABI 7500 Real-Time PCR System (Thermo Fisher Scientific, Waltham, MA, USA). The reaction mixture with a final volume of 20 μl contained 10 μl of ABI SYBR Green PCR Master Mix (Thermo Fisher Scientific), 1 μl each of 10 μM corresponding primers, and 1 μl of template DNA. All 1404 samples were first tested by a RT-PCR targeting *T. whipplei*-specific regions of the 16S–23S rRNA ITS that was demonstrated to be conserved in *T. whipplei* [6, 8]. For confirmation, the samples positive for the 16S–23S rRNA ITS were tested by the follow-up RT-PCRs targeting *rpoB*, *hsp65*, and *Dig15* (Table 1) [10, 11, 16]. Each RT-PCR was performed with a recombinant plasmid (pMG-Amp, Macrogen, Seoul, Korea) containing each target sequence as a positive control and distilled sterile water as a negative control, referring to the previous studies [10, 24]. Each RT-PCR was performed for 40 amplification cycles. The results of each RT-PCR were interpreted as positive when the following conditions were satisfied: 1) fluorescence from the target sequence exceeded the background signal; 2) the melting peak corresponded with the melting temperature (*T*_m_) of each positive control plasmid ± 1 °C; and 3) RT-PCR products were confirmed by electrophoresis in 2% agarose gels, staining with ethidium bromide, and detecting under UV light (data not shown). In the samples positive for the 16S–23S rRNA ITS, the presence or absence of *T. whipplei* was confirmed by direct sequencing analysis for the 16S–23S rRNA ITS (Fig. 1). Direct sequencing analysis was performed using the ABI 3730*xl* Analyzer (Thermo Fisher Scientific). The RT-PCR products were sequenced with the same forward primer used to amplify the 489-bp 16S–23S rRNA ITS sequence. BLAST searches were performed on the NCBI website (http://www.ncbi.nlm.nih.gov) to compare the sequences with those currently available in GenBank.

**Table 1:** RT-PCR targets and their primers for detecting *Tropheryma whipplei* in this study

Target	Primer sequence (5′–3′)		Product size, bp	Limit of detection, copies/μL	*T*_m_, °C
	Forward	Reverse			
16S–23S rRNA ITS	CCGGTGACTTAACCTTTTTGGAGA	TCCCGAGGCTTATCGCAGATTG	489	190	84
*rpoB*	CTCGGTGTTGATGTTGATCCAA	GCACCGCAACCTCGGAGAAA	109	1830	63
*hsp65*	CGCGAAAGAGGTTGAGACTG	ACATCTTCAGCAATGATAAGAAGTT	357	26	82
*Dig15*	TGTTTTGTACTGCTTGTAACAGGATCT	GATGATAGGAGGGATAGAGCAGGA	155	599	74

*ITS* intergenic spacer, *RT-PCR* real-time PCR, *T*_*m*_ melting temperature

### Statistical analysis

Based on the results of the follow-up RT-PCRs and sequencing, the prevalence of *T. whipplei* was estimated in all 1404 samples. All samples were divided into two groups according to gender (female, n = 654; male, n = 750) and 10 groups according to age in years: < 10 (n = 825), 10–19 (n = 221), 20–29 (n = 94), 30–39 (n = 30), 40–49 (n = 26), 50–59 (n = 56), 60–69 (n = 59), 70–79 (n = 49), 80–89 (n = 59), and ≥ 90 (n = 15). The differences in *T. whipplei* prevalence according to gender and age were compared using a chi-squared test. In addition, the prevalence of *T. whipplei* was estimated separately for pediatric samples in patients aged ≤ 19.

Based on the sequencing results for *T. whipplei* presence, the performance of follow-up RT-PCRs was evaluated using sensitivity, specificity, positive predictive value, negative predictive value, and accuracy in the samples positive for the 16S–23S rRNA ITS. The positivity of the follow-up RT-PCRs was compared using McNemar’s chi-squared test for paired proportions. The concordance and agreement were evaluated between the follow-up RT-PCRs. The concordance was calculated using the following equation: (the total number of samples with the same test results)/(the total number of samples). The agreement was evaluated using Cohen’s kappa (κ) with 95% confidence intervals (CIs) and interpreted as follows: ≤ 0.20, none; 0.21–0.39, minimal; 0.40–0.59, weak; 0.60–0.79, moderate; 0.80–0.90, strong; and > 0.90, nearly perfect [25]. Statistical analysis was performed using MedCalc Statistical Software (version 20.015; MedCalc Software, Ostend, Belgium). A two-sided *P* < 0.05 was considered statistically significant.

## Results

*T. whipplei* was detected in 1.4% of all samples (20 of 1404) (Table 2). The detection of *T. whipplei* was higher in samples from female than male, but there was no significant difference found between them (*P* = 0.23). Similarly, there were no significant differences noticed between the 10 groups divided by age (*P* = 0.20). In pediatric samples, *T. whipplei* was detected in 1.6% (17 of 1046), and it was detected significantly higher in children aged 6–19 than in younger children aged 1–5 (2.7% vs. 0.7%, *P* = 0.01).

**Table 2:** Prevalence of *Tropheryma whipplei* in the stools of Korean patients with diarrhea

Sample	Prevalence of *T. whipplei*, n (%)	*P* value
Total (n = 1404)	20 (1.4)	
Gender		
Female (n = 654)	12 (1.8)	0.23
Male (n = 750)	8 (1.1)	
Age, yrs		
< 10 (n = 825)	11 (1.3)	0.20
10–19 (n = 221)	6 (2.7)	
20–29 (n = 94)	2 (2.1)	
30–39 (n = 30)	0	
40–49 (n = 26)	0	
50–59 (n = 56)	1 (1.8)	
60–69 (n = 59)	0	
70–79 (n = 49)	0	
80–89 (n = 59)	0	
≥ 90 (n = 15)	0	
Pediatric, years (n = 1046)	17 (1.6)	
1–5 (n = 559)	4 (0.7)	0.01
6–19 (n = 487)	13 (2.7)	

Thirty-three samples were positive in the initial RT-PCR targeting the 16S–23S rRNA ITS (Table 3). Of these, 17 samples were positive in two or more of the follow-up RT-PCRs, and all were confirmed *T. whipplei*-positive with ≥ 98.9% identity by sequencing. Four samples were positive only in the RT-PCR targeting *Dig15*, and two of them were confirmed *T. whipplei*-positive with ≥ 99.6% identity by sequencing. One sample was positive only in the RT-PCR targeting *hsp65,* and it was confirmed *T. whipplei*-positive with 99.8% identity by sequencing. Eleven samples were negative in all follow-up RT-PCRs, and were confirmed *T. whipplei*-negative by sequencing.

**Table 3:** Number of samples positive or negative for RT-PCRs and sequencing

No. of sample	Initial RT-PCR (16S–23S rRNA ITS)	Follow-up RT-PCR			Sequencing	
		*rpoB*	*hsp65*	*Dig15*	Positive	Negative
11	P	N	N	N	0	11
9	P	P	P	P	9	0
7	P	N	P	P	7	0
4	P	N	N	P	2	2
1	P	N	P	N	1	0
1	P	P	N	P	1	0

There were 33 samples positive for the initial RT-PCR for the 16S–23S rRNA ITS

*ITS* intergenic spacer, *N* negative, *P* positive, *RT-PCR* real-time PCR

In the samples positive for the 16S–23S rRNA ITS (n = 33), the sensitivities of the follow-up RT-PCRs targeting *rpoB*, *hsp65*, and *Dig15* were 50.0%, 85.0%, and 95.0%, respectively. The specificities were 100.0%, 100.0%, and 84.6%, respectively, and the accuracies were 69.7%, 90.9%, and 90.9%, respectively (Table 4). The positivity rates of the follow-up RT-PCRs targeting *rpoB*, *hsp65*, and *Dig15* were 30.3%, 51.5%, and 63.6%, respectively. The RT-PCR targeting *rpoB* showed significantly lower positivity than those targeting *hsp65* and *Dig15* (*P* = 0.04, *rpoB* and *hsp65*; *P* < 0.01, *rpoB* and *Dig15*). However, there was no significant difference in positivity between the RT-PCRs targeting *hsp65* and *Dig15* (*P* = 0.22). The concordance and agreement were 72.7% and weak (κ = 0.46; 95% CI 0.19–0.73) between the RT-PCRs targeting *rpoB* and *hsp65*, respectively; 66.7% and minimal (κ = 0.39; 95% CI 0.17–0.63) between the RT-PCRs targeting *rpoB* and *Dig15*, respectively; and 81.8% and moderate (κ = 0.63, 95% CI 0.38–0.89) between the RT-PCRs targeting *hsp65* and *Dig15*, respectively.

**Table 4:** Performance of the follow-up RT-PCRs for detecting *Tropheryma whipplei* (n = 33)

Target	Sensitivity, % (95% CI)	Specificity, % (95% CI)	Positive predictive value, % (95% CI)	Negative predictive value, % (95% CI)	Accuracy, % (95% CI)
*rpoB*	50.0 (27.2–72.8)	100.0 (75.3–100.0)	100.0	56.5 (45.6–66.8)	69.7 (51.3–84.4)
*hsp65*	85.0 (62.1–96.8)	100.0 (75.3–100.0)	100.0	81.3 (60.4–92.5)	90.9 (75.7–98.1)
*Dig15*	95.0 (75.1–99.9)	84.6 (54.6–98.1)	90.5 (72.6–97.2)	91.7 (61.6–98.7)	90.9 (75.7–98.1)

*CI* confidence interval, *RT-PCR* real-time PCR

## Discussion

*T. whipplei* is commonly present in the intestine as its preferred niche [1]. Previous studies have suggested that stools have higher bacterial loads than saliva [13–15]. In Korea, a few studies have been conducted to detect *T. whipplei* and to estimate its prevalence in joint fluids, saliva, and gastric juices [12, 22, 23]; however, the prevalence has not been evaluated in stools. We focused on stools to estimate the prevalence of *T. whipplei* and performed the follow-up RT-PCRs using three target sequences and sequencing to confirm the presence of *T. whipplei*.

Similar to previous Korean studies conducted with saliva, *T. whipplei* was detected low in the stools of patients with diarrhea, with a prevalence of 1.4% [22, 23]. A recent study investigating the stools of patients with diarrhea for *T. whipplei* in three locations on different continents reported that its prevalence may differ between continents [26]. The prevalence of *T. whipplei* was 17.5% in South Africa, 15% in Singapore, and 3.3% in Germany, all higher than in Korea [26]. To date, Whipple’s disease has not been reported in Korea, and it has been reported very rarely in Japan [23, 27, 28]; it is in line with the low prevalence of *T. whipplei* found in our study. Although Whipple’s disease has never been reported, our results revealing the presence of *T. whipplei* in Korea support the hypothesis that this bacterium occurs naturally in humans [1, 14, 15]. In all samples, there was no significant difference in *T. whipplei* prevalence according to gender and age. When we focused only on pediatric samples, we observed a significantly higher prevalence in children aged 6–19 (Table 2). Similarly, the prevalence of *T. whipplei* was higher in children aged ≥ 5 years in Ghana and Laos [18, 21]. In contrast, the prevalence was higher in younger children aged 0–4 years in Senegal and Gabon [15, 17]. The prevalence in these countries was much higher than that in our study [15, 17, 18, 21]. Regional difference in prevalence may be due to different living conditions, such as hygiene conditions [17].

To exclude false-positive results determined by the PCR targeting the 16S rRNA sequence of *T. whipplei*, an additional PCR targeting more specific repetitive sequences is recommended [29]. Considering that only one RT-PCR targeting the 16S–23S rRNA ITS can produce false-positive results, the samples positive in the initial RT-PCR was tested with three follow-up RT-PCRs as confirmation tests. In 11 of 33 samples positive for the 16–23S rRNA ITS, all follow-up RT-PCRs were negative, and the sequencing showed no *T. whipplei*-specific sequence (Table 3). The RT-PCR targeting the 16S–23S rRNA ITS seemed to show high false-positive results in this study. This result can make the RT-PCR conditions used in this study seem to be rather unspecific. However, the conditions of all RT-PCRs were set through several pre-tests performed using serially diluted positive controls, referring to the previous protocols [8, 10, 16].

Not all *T. whipplei*-positive samples confirmed by sequencing were positive in all follow-up RT-PCRs. Several samples showed discrepant results between the follow-up RT-PCRs (Table 3). There was a difference in the performance of the follow-up RT-PCRs (Table 4). The RT-PCR targeting *Dig15* was the most sensitive, and that targeting *rpoB* was the least sensitive. The PCR targeting *rpoB* is known to be sensitive; its sensitivity was determined at 17.4 microorganisms per 5 μl suspension [16]. However, in this study, the sensitivity of the RT-PCR targeting *rpoB* was low similar to that of previous studies [10, 30]. The RT-PCR may lack sensitivity to determine the localized infection, which is particularly associated with a low copy number of *T. whipplei* in low-prevalence population [10, 13]. The low sensitivity of *rpoB* may also be associated with its relatively high limit of detection (Table 1). The specificity of the RT-PCR targeting *Dig15* was lower than those targeting *rpoB* and *hsp65*; the sequencing results of the two samples deemed positive by the RT-PCR targeting *Dig15* were negative for *T. whipplei*. The RT-PCRs targeting *hsp65* and *Dig15* were more reliable than that targeting *rpoB*. A few commercial kits have been developed for the research detection of *T. whipplei*, which use a single PCR target: LightMix Modular *T. whipplei* (TIB MOLBIOL, Berlin, Germany), which detects a 75-bp long fragment from a repeated genomic sequence and the BactoReal Kit *Tropheryma whipplei* (Ingenetix, Wien, Austria), which detects *hsp65*. The LightMix kit showed low sensitivity, and the BactoReal Kit was limited in that it could produce false-negative results and had lower sensitivity [10]. Our results revealing discrepancies and performance differences between the follow-up RT-PCRs imply that a multiplex PCR with more than two targets, including *hsp65* and/or *Dig15*, could help detect *T. whipplei* more accurately and reliably.

This study provides baseline data about the presence of *T. whipplei* in the stools of Korean patients with diarrhea because it was performed on a large cohort including all ages. In addition, positive controls that were not used for PCRs in previous Korean studies were used in this study [12, 22, 23]. Without positive controls for the PCRs, inaccurate results may have been obtained. Since it was difficult to obtain a standard strain of *T. whipplei*, we performed the RT-PCR by including recombinant plasmids as positive controls. On the other hand, this study has limitations. First, we included only patients with diarrhea in this study. For accurate evaluation, healthy individuals without diarrhea and other symptoms should also be included and compared with the patients with diarrhea. *T. whipplei* and diarrhea are significantly associated [15, 31]; therefore, the prevalence of *T. whipplei* in the stools of healthy individuals in Korea may be significantly lower than in patients with diarrhea. Moreover, most of the patients in this study were pediatric patients aged ≤ 19; thus, all subjects showed no normal distribution for prevalence estimation. Further studies would be needed in a cohort that evenly include subjects of various ages. Second, this study was conducted using the residual samples that were stored after the requested test was performed. The information of subjects could only be checked through a medical chart review. Since the information available in the medical charts was limited, we did not consider factors that could affect the prevalence estimation, such as what treatment procedures were taken before sampling. Antibiotics including ceftriaxone, meropenem, or doxycycline are used to treat Whipple’s disease [1]. In patients who took these antibiotics before sampling, the results for *T. whipplei* could be affected. Since the prevalence of *T. whipplei* varies from region to region, regional factors as well as antibiotics should be considered [15, 17, 18, 21, 26–28]. Third, although the total number of samples was large, the follow-up RT-PCRs were performed on the small number of samples that were deemed positive by the initial RT-PCR. Nevertheless, our results are likely to be reliable because they are similar to the results of a previous study with more samples [10]. It might be concerned that this study did not include an internal control for each RT-PCR, which could lead to false-negative results. However, the nucleic acid samples used in this study were the samples remaining after the requested PCR test was performed including the internal control. It could be considered that DNA was successfully extracted from each stool sample. Last, we did not consider the methodological difference in detecting PCR amplification. In more recent studies on the RT-PCR for *T. whipplei*, the bacterium-specific probes as well as SYBR green have been used [32–34]. The SYBR green dye can also bind to non-specific double-stranded DNA, which can lead to more false-positive results or lower sensitivity [35].

## Conclusions

This is the first study that estimated the prevalence of *T. whipplei* in the stools of Korean patients with diarrhea. This study demonstrated the presence of *T. whipplei* in the stools of Koreans, even though the bacterium was detected low. The RT-PCRs targeting *hsp65* and *Dig15* showed reliable performance, and a multiplex PCR including these targets is expected to be useful for detection of *T. whipplei* that is difficult to cultivate in clinical microbiology laboratories.

## Data Availability

The data used and analyzed during this study are available from the corresponding author on reasonable request.

## Abbreviations

*T. whipplei*: *Tropheryma whipplei*
PAS: Periodic acid-schiff
PCR: Polymerase chain reaction
ITS: Intergenic spacer
RT-PCR: Real-time PCR
*rpoB*: The β-subunit of RNA polymerase
*hsp65*: Heat shock protein 65
WiSP: Wnt1-inducible signaling pathway
*Dig15*: *Dig15* Gene segments from WiSP family protein
CAUMC: Chung-Ang University medical center
IRB: Institutional review board
*T*_m_: Melting temperature
κ: Cohen’s kappa
CI: Confidence interval
